# 胸腺瘤放疗的相关定义和报告指南

**DOI:** 10.3779/j.issn.1009-3419.2014.02.08

**Published:** 2014-02-20

**Authors:** Daniel Gomez, Ritsuko Komaki, James Yu, Hitoshi Ikushima, Andrea Bezjak, 北京大学肿瘤医院 付浩

**Affiliations:** 1 Department of Radiation Oncology, MD Anderson Cancer Center, Houston, Texas; 2 Department of Radiation Oncology, Yale University School of Medicine, New Haven, Connecticut; 3 Department of Radiation Oncology, Institute of Health Biosciences, University of Tokushima, Tokushima, Japan; 4 Department of Radiation Oncology, Princess Margaret Hospital, To-ronto, Ontario, Canada

放疗在胸腺肿瘤的治疗中仍然占有重要地位，但是很多细节没有明确的定义。例如，如何放疗、如何确定放疗范围、哪些患者需要放疗和如何报告放疗结果都没有统一的标准。ITMIG的成立，给解决这些问题创造了机会。但是先决条件是制定统一的定义和方法，使不同结果能被理解和比较，这也是本文的主旨。

## 1 方法

先由一个工作组（Daniel Gomez, Ritsuko Komaki, James Yu, Hitoshi Ikushima, and Andrea Bezjak）回顾相关文献和形成初步建议，再提交给下一个扩展工作组（Charles Tomas, Lynn Wilson, Gregory Videtic. James Metz, Harun Badakhshi, Clifton Fuller, Franc- oie Mornex, Conrad Falkson, David Ball, and Ken Rosenzweig）审议，之后将建议提交给ITMIG多学科组进一步讨论，形成初稿（包含推荐方法和标准操作流程）后分发给所有ITMIG成员更进一步讨论并反馈意见，最终版本经ITMIG批准并被采用。

## 2 基本定义

### 2.1 临床应用和放疗目的

胸腺瘤和其他胸腺肿瘤的主要治疗方法是手术。放疗主要用于术后，以降低纵隔复发风险。然而，根治性放疗也可以用于其他临床情况，例如肿瘤不可切除或经过术前化疗后仍然不可切除的患者。由于这些肿瘤大多数局限在胸腔，放疗范围应包括一个或多个胸腔结构，如纵隔、胸膜和心包，主要目的是控制肿瘤和消除肿瘤。然而，在复发病例中，放疗剂量和治疗目的可能不同，例如，对旨在改善症状而非根除肿瘤的患者可使用低剂量。因此，需要明确说明临床情况、放疗目的和范围，这样使不同研究之间的比较更有意义，以便于判断胸腺肿瘤的放疗疗效和制定进一步治疗方案。推荐定义见表 1。

**1:** 胸腺恶性肿瘤放疗报告指南 Summary of Reporting Guidelines and Data Fields for Thymic Malignancies Treated with Radiation Therapy

报告项目	描述内容
目的	姑息 术前(降期或缩小体积） 根治
	单一放疗
	同步或序贯化疗
	术后
	术后R0切除 术后R1切除 术后R2切除
放疗野	可见肿瘤及外放边界 瘤床及边缘
	累及区域以外的选择部位，如纵隔和淋巴结 胸膜转移部位 半胸腔
放疗剂量	起始日期和结束日期 放疗剂量，初始靶区(Gy) 分割剂量，初始靶区(Gy)
	局部推量放疗：是/否
	增量时间：序贯/同步 增加剂量(Gy) 增加分割剂量(Gy)
放疗技术	二维放疗 三维适形放疗 调强适形放疗 质子疗法 其它
放疗后局部复发	放疗野内 放疗野边缘 放疗野外
放疗毒副反应(CTC V4.0)	3级-5级：食管炎/心脏毒性/呼吸系统毒性/其它 最高毒性级别 毒副反应持续时间 剂量限制性毒副反应：是/否
CTC V4.0:通用毒副反应判定标准V4.0。 注：本表得到版权所有者© 2011 by the International Association for the Study of Lung Cancer复制许可。	

#### 2.1.1 临床应用

a) 术前——可行单独化疗或放疗，或者同期放化疗或序贯放化疗。

b) 术后——行术后放疗，肿瘤医生或文献作者应说明的切除情况，如完全切除（R0）、镜下肿瘤残余（R1）或肉眼残余（R 2），因为不同情况使用的放疗剂量不一样。如果术后既行放疗又行化疗，也应予以说明，因为这可能影响治疗毒性和结果。放疗范围包括部分或整个纵隔、心包、半胸腔，若行胸膜转移灶切除还应包括部分胸膜。

c) 根治性放疗（联合或不联合化疗）——如果未打算行手术切除，单放疗或放化疗应作为胸腺肿瘤的主要治疗。

d) 肿瘤复发放疗——需要详细说明复发区域和放疗类型，如体外照射、腔内放疗和术中放疗。

#### 2.1.2 目的

a) 根治——目的是根治肿瘤，例如长期疾病控制。

b) 姑息——目的是控制症状和/或缩小肿瘤体积，但不根除肿瘤。

#### 2.1.3 治疗范围

a) 可见肿瘤及边缘，如原发肿瘤或淋巴结。

b) 瘤床及边缘，根据术前和术后的影像和手术结果来确定，包括术中放置银夹的区域。

c) 超出肿瘤累及区域的选择性部位，如纵隔结构和淋巴结。

d) 胸膜转移部位（根治术后或根治、姑息治疗后，胸膜播散或种植）

e) 半胸腔（左侧或右侧）

### 2.2 放疗疗效评估

文章《胸腺恶性肿瘤预后标准评估方法》提出了预后评估的具体定义及其适用对象，同时该文章也提出了复发的相关定义（包括复发时间和局部、区域或远处复发）和讨论了如何评估疗效（化疗和放疗）^[1]^。简而言之，局部复发定义为肿瘤出现在胸腺瘤床处，区域复发是指发生在胸腔内，但与胸腺和原发胸腺肿瘤不相邻，远处复发是指区域以外复发，包括肺内结节。将来的研究都要根据这些定义对结果进行具体说明和报道。关于放疗需要具体说明的是，术后放疗后局部或区域复发是发生在放射野边缘还是完全在放射野之外（通过与线束边缘的关系来确定）。对此明确说明是很重要的，因为治疗计划经历了从广野的二维放疗（包含很大一部分纵隔）到三维适型调强放射治疗（减少了瘤床和前纵隔区域的照射范围）的转变，这种转变有潜在增加边缘和放射野之外治疗失败的风险。需要谨慎和详细报道复发部位与照射范围的关系，并收集这些数据来说明放疗技术的改变不会对胸腺肿瘤患者的预后造成不良影响。

## 3 放疗技术

### 3.1 放疗模拟定位

必须严格固定患者，最好是仰卧位，颈部稍微伸展，双上肢放在头部以上。这种体位适合共面和/或非共面照射角的多野照射技术。模拟CT扫描建议采用3 mm-5 mm的层厚。为更好的区分靶区和正常结构可以考虑使用静脉造影剂。如果可能的话可使用4维CT扫描，以便在制定计划时可评估靶区的运动情况^[2]^。如果没有4维CT扫描，肿瘤放疗医生可以通过以下几点来评估体内运动程度：（1）利用慢速螺旋CT采集整个呼吸过程中的图像，（2）采集最大吸气末和最大呼气末的CT图像，根据其差异可评估在呼吸过程中的运动幅度。CT扫描结合PET扫描可以作为治疗计划的辅助手段^[3]^。

以下靶区需要报道：

a) 可见肿瘤靶区（GTV）——主病灶。

b) 临床靶区（CTV）——GTV加镜下浸润区域或镜下浸润高危区域。在以前，CTV覆盖了整个纵隔，包括了术前病灶范围。然而随着图像更清晰，包括广泛应用CT模拟定位，使得一个更局限的CTV能包括术后的高危区域，这些局域是根据术前影像和术中发现确定的，包括外科留置银夹的高危区域。对受累部位和边缘或之外的区域进行选择性放疗时，如淋巴结或纵隔，治疗医生要加以说明。

c) 内靶区（ITV）——CTV加体内运动。

d) 计划靶区（PTV）——ITV加患者摆位误差。

对于术后患者，靶区应该结合患者术前术后影像勾画。应该用术前的影像评估肿瘤范围。

靶区边界勾画取决于模拟定位技术，如4D CT模拟定位和每日的图像引导。如果没有这些技术，根据肿瘤的位置（例如肿瘤位置越靠下，活动度越大）和治疗医师经验可以考虑扩大边缘范围。推荐以下边界范围：

a) GTV到CTV边界：0.5 cm-1 cm

b) CTV到PTV边界，在没有4D CT和每日的图像引导情况下：1 cm-1.5 cm

c) ITV到PTV边界，有4D CT模拟定位但是没有每日的图像引导：0.5 cm-1 cm

d) ITV到PTV边界，有4D CT模拟定位和每日的图像引导：0.5 cm。

ITMIG的一个由放疗/肿瘤放疗专家组成的工作组正试图制定一套图谱，以指导靶区的勾画。

### 3.2 放疗计划

既往胸腺瘤的放疗采用的是二维和简单的射野设计。然而，由于近年放疗技术的进步，强烈推荐适形放疗技术，可以保护周围的纵隔结构。这些技术包括三维适形放疗和调强适形放疗。

“三维适形放疗”定义为使用多波束和构建剂量体积直方图来评估分布在肿瘤和正常结构的剂量（特别是以下器官剂量：食管，心脏，肺和脊髓）。“调强适形放疗”（Intensity-modulated radiation therapy, IMRT）定义为类似三维适形放疗，通过多波束治疗，另外它可以在治疗中改变放射线的影响和逆向调强治疗（即在设计治疗射束前就限定好正常组织的受量）。IMRT技术相比三维适形放疗具有高适形度的优势。在几项肺癌的放射量测定研究和最近评估三维适形放疗与IMRT治疗的预后和毒性的临床研究中，都显示IMRT治疗优于三维适形放疗^[4]^。尽管没有在胸腺瘤中直接比较，也可以推测出调强适形放疗在该肿瘤中的优势，因为胸腺瘤也发生在胸腔，周围有相同的重要器官。NCCN胸腺肿瘤治疗指南强调，如果使用IMRT，治疗医生应该根据美国肿瘤放疗协会的指南优化治疗计划。放疗技术的描述见表 1。

### 3.3 胸腺瘤的放射剂量测定参数

表 2概括了胸腺瘤患者正常结构组织放射剂量测定参数，这些参数来自最近临床指南中的正常组织定量分析，并应用于胸部放疗所有的部位^[5]^。治疗时间、分割剂量、射线能量、放疗野形态、总剂量和剂量体积直方图参数都应该详细描述。

**2:** 胸腺恶性肿瘤放疗中限定剂量及报告方式 Dosimetric Constraints to be Used and Reported in the Treatment of Thymic Malignancies^[5]^

	单纯放疗	放疗联合化疗	术前放疗联合化疗
脊髓^a^	D_max_ < 45Gy	D_max_ < 45Gy	D_max_ < 45Gy
肺^b^	MLD≤20 Gy	MLD≤20 Gy	MLD≤20 Gy
	V_20_≤40%	V_20_≤35%	V_20_≤30%
		V_10_≤45%	V_10_≤40%
		V_5_≤65%	V_5_≤55%
心脏	V_30_≤45%	V_30_≤45%	V_30_≤45%
	平均剂量 < 26 Gy	平均剂量 < 26 Gy	平均剂量 < 26 Gy
食管	D_max_≤80 Gy	D_max_≤ < 80 Gy	D_max_≤80 Gy
	V_70_ < 20%	V_70_ < 20%	V_70_ < 20%
	V_50_ < 50%	V_50_ < 40%	V_50_ < 40%
	平均剂量 < 34 Gy	平均剂量 < 34 Gy	平均剂量 < 34 Gy
肾^c^	20 Gy < 32%的单侧肾	20 Gy < 32%的单侧肾	20 Gy < 32%的单侧肾
肝	V_30_ ≤40%	V_30_ ≤ 40%	V_30_ ≤ 40%
	平均剂量 < 30 Gy	平均剂量 < 30 Gy	平均剂量 < 30 Gy
^a^治疗中应考虑脊髓的放疗范围，因为随着范围的扩大对脊髓的损伤也随之增加。当PTV非常接近脊髓时（< 1 cm), 如肿瘤侵犯脊柱，脊髓接受的放射剂量高于推荐剂量。但是，即使靶区很小，脊髓的放疗剂量也不应超过60Gy。高分割剂量或高日接受放疗剂量会降低患者耐受性，若分割剂量为3 Gy, 那么脊髓的限制剂量应约为40 Gy。 ^b^V_20_=有功能的肺接受20 Gy及以上剂量的体积。对于放疗前行单侧全肺切除的患者，推荐剂量为MLD < 8 Gy、V_20_ < 10%和V_5_ < 60%。注意，对于肿瘤完全切除的患者没有GTV, 故肺部限制剂量就代表了全肺，而非用全肺剂量减去CTV。 ^c^如果肾脏需行高剂量大范围放疗，应行肾功能扫描。 MLD:平均肺剂量；D_max_:最大剂量；PTV:计划靶区；CTV:临床靶区；GTV:肿瘤靶区。 注：本表得到版权所有者© 2011 by the International Association for the Study of Lung Cancer复制许可。			

## 4 胸腺瘤术后放疗

### 4.1 胸腺瘤R0切除术后放疗

术后放疗应该在术后3个月内进行。如果术后初次影像学提示没有肿瘤残余，也没有建议行放疗，但是随后的影像学发现肿瘤复发，这时对复发病灶进行的放疗不能称为术后放疗。发表的文章中应该记录放疗目的，如“复发病灶放疗”。我们建议术后辅助放疗的最小剂量为40 Gy，1.8 Gy-2 Gy分割，即使没有肿瘤残余。不管哪种情况，都要像表 1中那样报告剂量，还要记录开始日期、结束日期、总剂量、分割剂量、瘤床剂量和推量剂量。半胸腔剂量不同于手术野的治疗剂量，这个问题在后面讨论。当肿瘤完全切除，术后的最小放射范围应该包括术前病灶范围（根据术前影像提示）和术中发现的高危区域。如果没有其它地方需要治疗，这个区域应报告为“瘤床及其边缘”。如果对选择性部位治疗，包括整个纵隔或选择性淋巴结，这个区域应报告为“瘤床以外选择性区域”（表 1）。如果淋巴结活检证实转移，淋巴结放疗就不是选择性的。注意，任何治疗的病变区域应定义为“受累区域”、或“瘤体及其边缘”，记录这些区域是否包括原发部位、受累淋巴结、或胸膜种植灶切除部位。

### 4.2 R1切除术后放疗

R1切除术后放疗的时机跟R0切除是一样（术后3个月内），但是需要注意的是，必要联系外科医生和病理科医生明确残余灶的解剖部位。如果在切除过程中标本按照ITMIG建议处理，这些区域都标记有银夹，很容易辨认^[6]^。其次，如果术后影像没有发现肿瘤残余也没有进行放疗，但是在之后的影像检查中又发现肿瘤并建议放疗，那么放疗目的应该是针对“复发”，而不能称为术后放疗。应注意，不要将术中用的止血材料误认为复发灶。如果术后影像发现没有肿瘤残余，也推荐放疗，但是放疗前影像检查发现又快速生长出来，这种情况应该定义为术后“肿瘤肉眼残余”，记录为R2。对于术后放疗，认为合适剂量应为40 Gy-64 Gy，并根据表 1报告放疗剂量。由于剂量范围大，需要考虑正常组织的耐受剂量。再次，半胸腔剂量是一个单独的问题，不同于手术区域的治疗，这在后面有讨论。当肿瘤完全切除，最小放射野应包括术前受累区域、手术标记区域、外科医生和病理科医生指示为阳性切缘或潜在镜下肿瘤残余的区域。放疗野应按以下标准报告，包括“瘤床及其边缘”或“受累区域之外选择性部位”的分类。如果活检证实淋巴结为转移，对其放疗不再是选择性。

### 4.3 切除术后放疗

R2切除术后放疗的时机和R0、R1切除是一样的，一般也是术后3个月内。但是，如果一个R2切除的患者由于疾病表现活跃而行术后化疗序贯放疗，或者化疗序贯放化疗，那么手术到术后放疗的时间间隔可能超过3个月，而且放射治疗应描述为“根治性”目的。同样的，如果术后没有计划放疗，而是用了化疗，肿瘤没有得到控制，这时再行放疗也应定义为根治性放疗，而不是“术后放疗”。放射剂量低于54 Gy对肉眼残余病灶是不够的。最小放疗野应包括，术后影像检查中肿瘤范围和术中银夹标记区。放射野应按照下面报告，包括分为“肿瘤及其边缘”、“受累区域之外选择性部位”和/或“胸膜转移部位”。同样，如果活检证实淋巴结转移，该淋巴结不再是选择性的淋巴结（表 1）。

## 5 术前放疗的定义和报告指南

术前化疗、放疗或联合放化疗已被证实是对局部晚期疾病有效的治疗方案。术前治疗后，需要在治疗完成的3周-6周内通过影像学评效，如增强CT。如果评效后患者适合手术，应行手术切除，然后根据前述的指南评估术后放疗。如果患者术前做了放疗，在以后放射治疗中都要考虑术前的放疗剂量，而且要结合正常组织的耐受剂量制定一个“综合治疗计划”。这个综合计划既要考虑术前放疗剂量也要考虑目前放疗剂量，需要注意的是，这些放疗剂量不能简单相加，故常常无法准确定量。如果经过评估后，患者不适合手术，需要考虑按照以下根治性放疗指南进一步行放疗，也应该制定综合治疗计划。如果之前行了放射治疗，那么要限制进一步放疗的放射剂量。

在术前，放疗野的界定和报告应根据前面提到指南和尽可能多的使用影像图像，包括GTV、镜下侵犯边缘、体内运动和患者摆位（CTV、ITV和PTV），半胸腔放疗技术在后面描述。强烈推荐使用高清的增强CT扫描。术前放疗剂量应在40 Gy-64 Gy。对于术后放疗，根据前面提到的指南决定剂量，并且根据切除情况、术中发现和正常组织耐受剂量来调整放射剂量。放射剂量和放射野报告见表 1。

## 6 胸腺瘤根治性放疗

根治性放疗定义为一种控制疾病的独立的局部治疗手段，尽管放疗经常联合全身化疗。适用于以下胸腺瘤患者：（1）不能手术的患者或（2）术前治疗后不可切除的患者。“不能手术”定义为患者不能耐受手术切除，不是因为肿瘤恶性程度，而是因为年龄、合并症和身体状况等因素。前面提到，“根治性放疗”的定义也能用于R2切除的患者，因此，放疗可以作为一种主要的以根治为目的的局部治疗手段。放疗野报告见表 1，要覆盖GTV和适当的镜下浸润边缘、体内运动和患者摆位变化。建议使用高清胸部增强CT扫描、根治性放疗剂量不得低于54 Gy。

## 7 半胸腔放疗在胸腺瘤治疗中的地位

“半胸腔放疗”是指除了PTV之外，还包括整个一侧胸腔。半胸腔放疗的理论基础是有研究表明胸腺瘤有潜在胸膜播散可能，而且认为低剂量放疗可以消灭该区域肿瘤^[8]^。半胸腔放疗的合适放疗野用前后放射技术界定：上，胸廓入口，或肿瘤的最高点；下，膈；外侧，距皮肤1 cm；中，对侧椎体^[8]^。然而，应该注意的是，这项技术的靶区是胸膜，而不是肺实质，尽管目前还没有研究证实适形放疗技术对于这种情况可以保护肺组织，但这必将是研究的焦点，以评估这种方法（仅针对危险区放疗，而将正常组织剂量降到最低）的安全性和有效性。半胸腔放疗已用于Ⅲ期-Ⅳa期胸腺瘤^[6, 7]^。半胸腔放疗对于分期较早的胸腺瘤（Ⅱ期-Ⅲ期）剂量范围是10 Gy-17 Gy，7次-16次分割。高危区域增量至50 Gy-70 Gy（1.8 Gy-2 Gy分割），Ⅳa期胸腺瘤增量至45Gy-54Gy。放疗靶区按前面概述的方法界定（GTV、CTV、ITV和PTV）。关于半胸腔放疗报告，不仅要报告半胸腔区域放射剂量，还要报告初始靶区范围和具体分割，对于肿瘤受累区域或高危区域的放疗应报告增量（表 1）。

## 8 放疗后复发

尽管适形放疗技术有诸多优点，尤其是调强适形放疗，但一个潜在的缺点是可能会漏掉某个放疗区域。这种复发形式可以定义为放疗野之外的疾病进展，命名为“照射野外复发”，或者定义为高剂量衰减区域疾病进展，命名为“边缘缺失”。边缘缺失的概念在其他恶性肿瘤中已经详细阐明，如头颈部肿瘤，但是在文献中没有统一的严格定义^[10-12]^。我们建议定义为局部复发肿瘤中心位于处方剂量（≥100%处方剂量）和放射野边缘（≥50%剂量）之间的区域。放疗野外复发定义为局部复发肿瘤中心位于放射野边缘之外或50%等量线之外。因此，局部复发的报告分三类：放射野内、边缘复发和放射野外复发。我们知道，在界定剂量衰减等量线时常常存在一定程度的主观性，因此使用一个合理的计量数字来界定放射野的复发可以避免这种主观性。这些定义将指导数据的收集和提高靶区勾画的精度。

## 9 胸腺瘤治疗的毒性报告

所有胸腺肿瘤放疗相关毒性应根据表 1报告。相关毒性包括：食管炎、肺炎、呼吸困难、皮肤炎和心脏毒性，如心包炎、心率失常和冠状动脉疾病。因为胸腺肿瘤的惰性生物学行为和相对高的长期生存率，急性毒性（肿瘤放疗组定义为开始放疗3个月内）和慢性毒性（3个月外的副作用）都要报告。放射肿瘤协作组标准和常见治疗不良反应标准都可以使用，但是治疗医生需要具体说明引用的毒性评分系统和版本。

尽管已经发表了很多关于胸腺肿瘤的放疗指南，但以上建议可使不同研究结果具有可比性，通过提供基本的病历文件帮助肿瘤放疗医生进行回顾性和前瞻性研究。遵循本文提供定义的最终目标是在制定治疗规范时将模糊性和不统一性降到最低，最终建立一个连续数据库，使数据能够很容易的被提取和更新，并且这些指南中的放疗术语不受地域、治疗水平和报告日期的影响。如果能达到这个目标，将会加速研究进展和提高研究的准确性，从而提高放疗对这种少见肿瘤的安全性和疗效。
